# Desmin variants: Trigger for cardiac arrhythmias?

**DOI:** 10.3389/fcell.2022.986718

**Published:** 2022-09-09

**Authors:** Wei Su, Stan W. van Wijk, Bianca J. J. M. Brundel

**Affiliations:** ^1^ Physiology, Amsterdam UMC Location Vrije Universiteit Amsterdam, Amsterdam, Netherlands; ^2^ Amsterdam Cardiovascular Sciences, Heart Failure and Arrhythmias, Amsterdam UMC Location Vrije Universiteit Amsterdam, Amsterdam, Netherlands

**Keywords:** desmin, cardiac arrhtyhmias, cardiac conduction system, desmosomes, *DES* gene variants

## Abstract

Desmin (*DES*) is a classical type III intermediate filament protein encoded by the *DES* gene. Desmin is abundantly expressed in cardiac, skeletal, and smooth muscle cells. In these cells, desmin interconnects several protein-protein complexes that cover cell-cell contact, intracellular organelles such as mitochondria and the nucleus, and the cytoskeletal network. The extra- and intracellular localization of the desmin network reveals its crucial role in maintaining the structural and mechanical integrity of cells. In the heart, desmin is present in specific structures of the cardiac conduction system including the sinoatrial node, atrioventricular node, and His-Purkinje system. Genetic variations and loss of desmin drive a variety of conditions, so-called desminopathies, which include desmin-related cardiomyopathy, conduction system-related atrial and ventricular arrhythmias, and sudden cardiac death. The severe cardiac disease outcomes emphasize the clinical need to understand the molecular and cellular role of desmin driving desminopathies. As the role of desmin in cardiomyopathies has been discussed thoroughly, the current review is focused on the role of desmin impairment as a trigger for cardiac arrhythmias. Here, the molecular and cellular mechanisms of desmin to underlie a healthy cardiac conduction system and how impaired desmin triggers cardiac arrhythmias, including atrial fibrillation, are discussed. Furthermore, an overview of available (genetic) desmin model systems for experimental cardiac arrhythmia studies is provided. Finally, potential implications for future clinical treatments of cardiac arrhythmias directed at desmin are highlighted.

## Introduction

For a healthy function of the heart, the crucial importance of intermediate filament (IF) proteins to maintain balanced communication within and between neighbouring cardiomyocytes has been recognized (Hnia et al., 2015; Henning and Brundel, 2017; Brodehl et al., 2018). Desmin is a key IF subunit expressed in specialized cardiac cell subpopulations related to the cardiac conduction system, including the sinoatrial node (Mavroidis et al., 2020), atrioventricular node (Benvenuti et al., 2012), and His-Purkinje system (Yuri et al., 2007). As such, desmin controls the structural and mechanical integrity of the contractile apparatus and is involved in the conduction of electrical signals within the heart (Tsikitis et al., 2018).

The important function of desmin in the cardiac conduction system is related to the distinctive property of desmin to form networks that connect and anchor various cell structures and organelles including desmosomes, costameres, Z-bands, the cytoskeleton, mitochondria, and nuclei (Henning and Brundel, 2017). In addition, desmin binds to various proteins within cardiomyocytes, modulating a variety of (Henning and Brundel, 2017) signaling pathways to maintain a healthy cardiomyocyte function. As a consequence, variations in the desmin (*DES*) gene have been reported in a number of cardiac diseases including atrial and ventricular arrhythmias, as well as hypertrophic-, restrictive-, dilated-, and non-compaction cardiomyopathy (Protonotarios et al., 2021). Although various papers describe the role of *DES* variants underlying cardiomyopathies, limited information is available on the molecular origin of *DES* variants, loss of expression, and the onset of cardiac arrhythmias, including atrial fibrillation (AF). As cardiomyocytes within the cardiac conduction system are particularly prone to age-related desmin dysfunction and consequently structural and functional impairment, desmin dysfunction increases the likelihood of arrhythmias and the requirement for pacemaker implantation in the growing aging population (Goldfarb et al., 2004). This review will provide up-to-date insight into the molecular and cellular mechanisms of desmin to underlie a healthy cardiac conduction system and how impaired desmin triggers cardiac arrhythmias, including AF. Furthermore, an overview of available genetic desmin model systems for experimental cardiac arrhythmia is provided. Finally, potential implications for future clinical treatments of cardiac arrhythmias directed at desmin are discussed.

## Desmin expression patterns during cardiac development

The distribution pattern of high desmin levels in the heart elucidates its role in the cardiac conduction system (Yamamoto et al., 2011). Immunohistochemical staining of human embryonic hearts described the spatial expression of desmin at different developmental stages. According to Carnegie stages, desmin is first expressed in the myocardial wall of the atrioventricular canal and the upper region of the primary ring at stage 11. During stage 12 through stage 13 of cardiac development, desmin is expressed in the primordium of the sinus node and right venous valve. When septation of the heart is almost complete, at late embryo stage 20, desmin is widely expressed in the primordium of the atrioventricular node, the atrioventricular bundle, the bundle branches, and at the entire ventricular trabeculations (Liu et al., 2020). The distribution of desmin throughout the conduction system and the key role of the desmin network in the maintenance of cardiomyocyte structure and mechanical function provides a direct morphological basis for investigating the mechanism of arrhythmogenesis caused by desmin impairment. This is supported by previous studies, which showed that genetic variations of *DES* and loss of desmin expression trigger cardiac arrhythmia (Liu et al., 2020).

## Desmin network and molecular interaction partners

**FIGURE 1:**
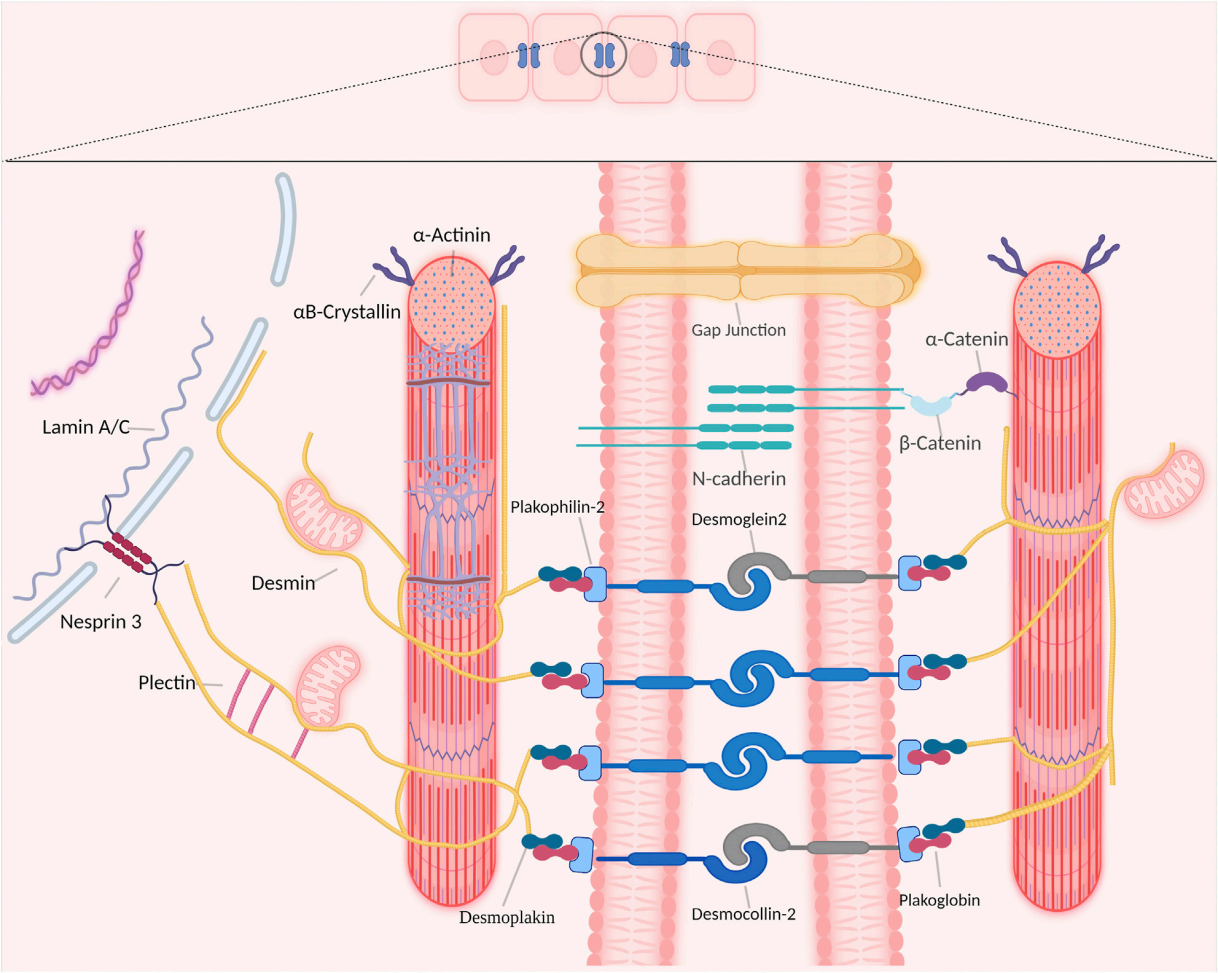
Overview of desmin network in and between cardiomyocytes. Desmin interacts with different organelles (including nucleus and mitochondria) and connects the entire contractile apparatus to different membranous compartments (such as sarcolemma, costameres and desmosomes of the intercalated disks) to form a continuous cytoskeletal intermediate filaments network.

Desmin is abundantly expressed in cardiomyocytes and represents one of the type III intermediate filaments. The important function of desmin in the cardiac conduction system is related to the distinctive property of desmin to form intra- and intercellular networks by connecting and anchoring various cytoskeletal structures and organelles, including desmosomes, mitochondria, nuclei, costameres, and Z-bands (Figure 1) (Herrmann and Aebi, 2004; Brodehl et al., 2018).

In cardiomyocytes, the desmin network plays an important role in striated myocardium development and maintenance by integrating and coordinating most cellular components necessary for proper mechanochemical signaling, organelle cross-talk, energy production, and trafficking processes required for proper tissue homeostasis (Capetanaki et al., 2015). Desmin interacts directly with various proteins within cardiomyocytes, as such desmin modulates a diversity of signaling pathways to maintain a healthy cardiomyocyte function. Desmin interacts directly with other members of the intermediate filaments family, costameres, cytolinkers bridging organelles and cytoskeleton, and the LINC-complex protein nesprin-3 that is present in the nuclear membrane (Figure 1) (Hol and Capetanaki, 2017). In addition, desmin binds indirectly to posttranslational modifications and signaling pathways that are important for proper skeletal or cardiac muscle functions (Figure 1).

Although it has been thoroughly described that desmin interacts with mitochondria, the nature of this interaction is not fully understood. Binding can be indirect through desmin-associated proteins, such as plectin. Moreover, the direct binding of desmin with the lipid phosphatase myotubularin has been shown to regulate mitochondrial dynamics, morphology, and function (Hnia et al., 2011). However, recent data shows that mitochondria can directly interact with desmin *in vitro* (Dayal et al., 2020). Mitochondrial function and structure abnormalities seem to be the earliest detected defects in desmin knockout cardiomyocytes. These defects include morphological aberrations in the form of mitochondrial swelling, increased mitochondrial size, disrupted cristae structure, loss in respiration, abnormal activation of mitochondrial permeability transition pore (mPTP), and dissipation of the mitochondrial membrane potential (Diokmetzidou et al., 2016). These alterations are known to play a role in cardiac arrhythmias, therefore a possible association between desmin disruption and the onset of cardiac arrhythmias has been suggested (Rutledge and Dudley, 2013; Pool et al., 2021).

**FIGURE 2:**
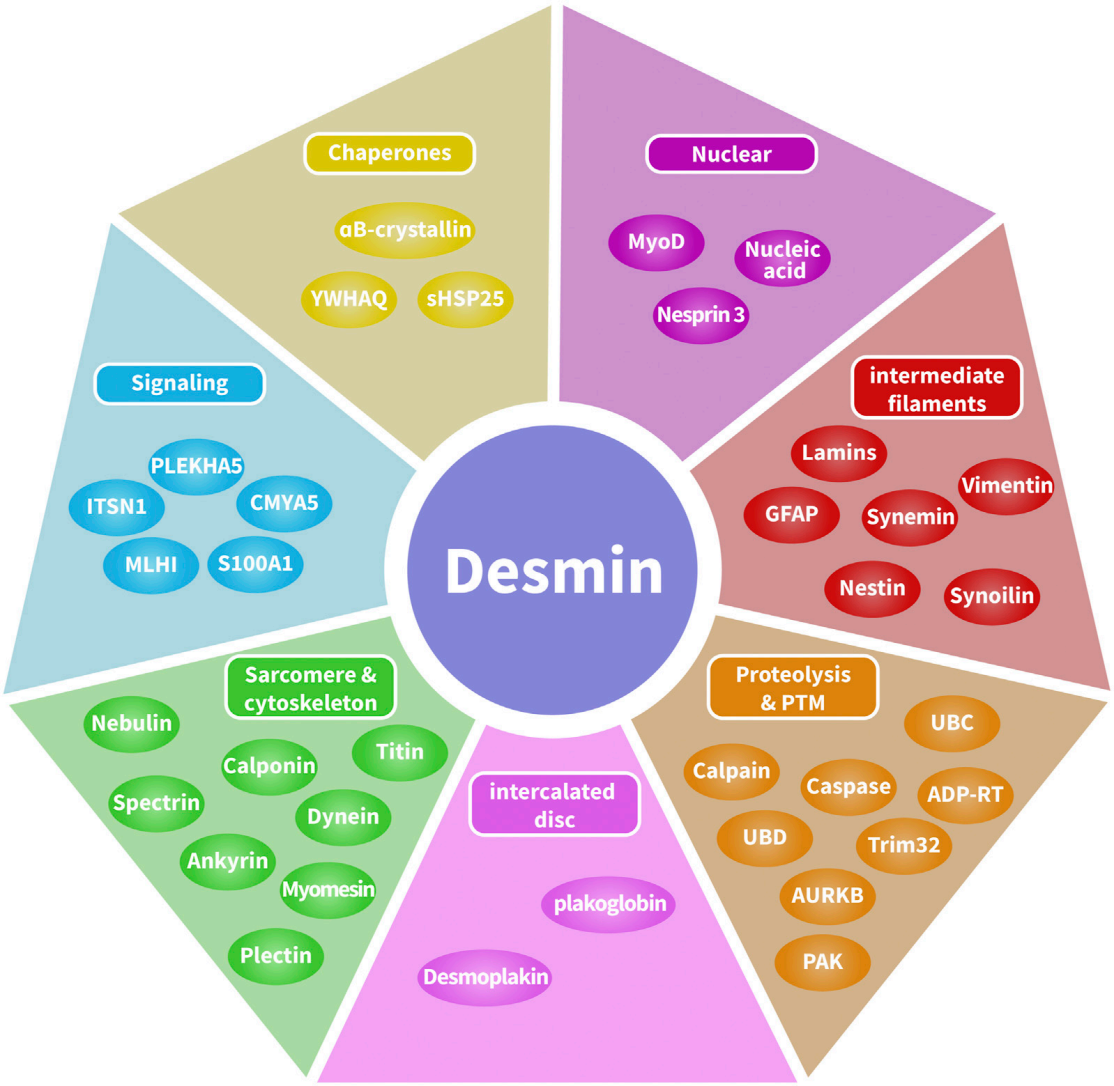
Desmin interactome map. Desmin-associated proteins include chaperones, DNA-related proteins, intermediate filaments, proteolysis and post-translational modifications, cytolinkers bridging sarcomeres and the cytoskeleton, and cellular signaling. Desmin-associated proteins are localized in multiple compartments, including costameres, contractile apparatus, intercalated discs, lysosomes, mitochondria, and the nucleus.

Recently, the various binding partners of desmin have been elucidated (Hnia et al., 2015). Based on published data (Hnia et al., 2015; Hol and Capetanaki, 2017) and interaction databases BioGRID^4.4^, the interactome of desmin binding partners has been depicted (Figure 2). Here, we introduce three categories of desmin protein partners in detail: intermediate filaments, intercalated discs, and αB-crystallin.

### Intermediate filaments

In muscle cells, desmin interacts with type III intermediate filament protein vimentin (Shahraz et al., 2017) and syncoilin (Poon et al., 2002), type IV intermediate filament protein synemin (Granger and Lazarides, 1980) and nestin (Cízková et al., 2009), and type V intermediate filament protein lamin (Cartaud et al., 1995). Lamins consist of two types, type A and type B, according to their structural similarities and isoelectric points. Lamin type A consists of lamin A and lamin C, both of them transcribed from *LMNA* and turned into lamin A and lamin C by alternative splicing. Lamin A/C is located in the nuclear interior. Lamin B is transcribed from *LMNB*, and along with heterochromatin, is anchored to the inner surface of the nuclear membrane by the lamin B receptor. Lamin B has been suggested to be a direct binding partner of desmin (Georgatos and Blobel, 1987). Interestingly, a desmin depletion leads to infolding of the nuclear envelope, loss of the nuclear integrity, increased amount of DNA damage, and diminished contractile function (Heffler et al., 2020). Similarly, the anchoring of desmin to the nucleus is lost in a Lamin A/C (*Lmna* ^−/−^) knockout mice model mice, which causes disorganization of the desmin (Nikolova et al., 2004). Furthermore, a known pathogenic lamin A/C variations can lead to desmin dysfunction as was shown in a cardiomyopathy *Lmna* ^*H222P/H222P*^ mouse model. Disturbance in the nuclear lamina introduced due to the *Lmna* variation leads to mislocalization of desmin at the Z-disk and intercalated disk, and desmin aggregate formation. Interestingly, overexpression of the small heat shock protein αB-crystallin or reduction of endogenous desmin, improved *Lmna* ^*H222P/H222P*^ cardiac function and pathology (Galata et al., 2018). Syncoilin is found at the neuromuscular junction, sarcolemma, and Z-lines, which are involved in the anchoring of the IF network at the sarcolemma and the neuromuscular junction. The dysfunction of syncoilin may result in the disruption of the IF network (Poon et al., 2002). Vimentin is expressed in mesenchymal cells, vimentin together with desmin and nestin play a significant role in the construction and restoration of skeletal myofibers (Sjöberg et al., 1994).

### Intercalated discs

Desmin interacts with the desmosomes of the intercalated discs through desmoplakin (Capetanaki et al., 2015). Intercalated discs consist of three types of cellular junctions: adherent junctions, desmosomes, and gap junctions. Intercalated discs are structures that connect adjacent cardiomyocytes, which are crucial for cell-cell mechanical and electrical connections, and as such are fundamental for cardiac function. In desmin knockout mice, changes in the morphology of intercalated discs were observed (Thornell et al., 1997). By immunohistological analysis of cardiac tissue heterozygous for the *DES* p.A120D mutation, Brodehl et al. (2013) found that desmin localization is completely lost at the intercalated discs. An impaired desmin network may slow electrical conduction, enhance conduction heterogeneity, and predispose patients to develop reentrant arrhythmias (Capetanaki et al., 2015).

### αB-crystallin

Transient transfection of H36CE cells with the small heat shock protein αB-crystallin (encoded by *CRYAB*) indicate that αB-crystallin and desmin form a functional complex (Elliott et al., 2013). The association of desmin with the αB-crystallin, and the fact that both the *CRYAB* p.R120G variant and *DES* variants lead to cardiac arrhythmias in mice (Jiao et al., 2014), and *CRYAB* variant p.D109G and *DES* variants lead to restrictive cardiomyopathy (Brodehl et al., 2017), suggest a potential compensatory interplay between the two in cardioprotection. Both proteins co-localize at the mitochondria-SR contact sites (Diokmetzidou et al., 2016), and disruption of the mitochonrida-SR contact sites has been suggested to underlie AF (Henning and Brundel, 2017; Wiersma et al., 2017; Li et al., 2019). Overexpression of αB-crystallin in desmin-deficient mice hearts ameliorates all mitochondrial defects and improves cardiac function significantly (Diokmetzidou et al., 2016).

## Desmin variants and clinical arrhythmias

Because desmin is located in different human tissues, the clinical phenotypes associated with *DES* variants are diverse. So far, over 60 different pathogenic *DES* variants have been described (Hnia et al., 2015). Most patients with *DES* variants develop combined skeletal and cardiac myopathy.

**FIGURE 3:**
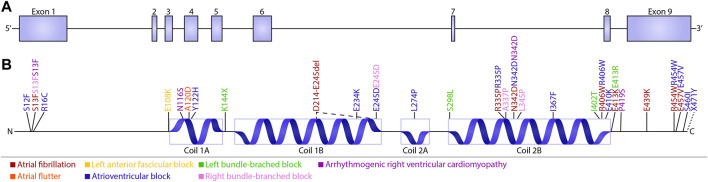
Schematic overview of cardiac conduction defects and arrhythmias associated with *DES* variants. **(A)** Schematic overview of the *DES* gene consisting of nine exons. **(B)** Schematic domain organization of desmin and the localization of the known conduction defects and arrhythmias associated with *DES* variants. Variants are subdivided into five groups depending on their related phenotype: Atrial fibrillation (brown), Left anterior fascicular block (yellow), Left bundle-branched block (green), Arrhythmogenic right ventricular cardiomyopathy (purple), Atrial flutter (orange), atrioventricular block (blue), and Right bundle-branched block (pink).

A limited amount of variants in desmin genes have been identified that affect exclusively cardiac function (for details, see Table 1). A meta-analysis including 40 different *DES* variants revealed that 50% of the carriers developed cardiomyopathy and around 60% cardiac conduction disease (CCD) or arrhythmias, with the atrioventricular block (AVB) as an important hallmark (van Spaendonck-Zwarts et al., 2011). *DES* variants with a pathogenic single nucleotide polymorphism located in coil 2B or near the carboxyl terminus of the gene, usually result into missense mutations that associate with conduction diseases and arrhythmias (Figure 3; Table 1). Moreover, several studies showed that single nucleotide polymorphisms (SNPs) around *DES* c.375G leads to a splice variant which excludes exon 3 (p.D214-E245del) (Ojrzyńska et al., 2017; Fan et al., 2019; Brodehl et al., 2021; Chen et al., 2021). The carboxyl tail of desmin has previously been suggested to play an important role in filament-filament interactions, and variations lead to filament flexibility and stiffening of the filament. Furthermore, Bär et al. (2010) suggest that variations in the carboxyl tail lead to impaired cellular mechanosensing and intracellular mechanotransduction (Bär et al., 2007; Bär et al., 2010). The rod part of desmin is made up of four coil domains and consists of a heptad-repeat arrangement which enables desmin to form stable parallel, two-stranded α-helical coiled-coil dimers. Notably, the end of the coil 2B region of desmin contains the “IF-consensus” motif: ‘“TYRKLLEGEESRI” (amino acid 404–416) which is important in filament assembly, stability, and contains multiple pathogenic variants (Figure 3; Table 1) (Bär et al., 2004; Herrmann and Aebi, 2004). So, gene variants located in coil 2B and/or carboxyl terminus of desmin are associated with clinical arrhythmias. As these locations are involved in filament stability and interactions, loss in filament network may trigger *DES* variant-induced cardiac arrhythmias. In addition, a previous study showed that *DES* p.N116S, a variant located in the conserved IF ‘LNDR’-motif which is located in the coil 1A region, leads to disturbed desmin filament formation and fuels aggresome formation (Figure 3).

**TABLE 1:** Overview of different types of cardiac conduction disease and arrhythmias related to *DES* variant carriers.

Cardiac arrhythmia	*DES* variant	Categorization	References
AF	p.R355P	Pathogenic	Wahbi et al., 2012
AF	p.R406W	Pathogenic	Wahbi et al., 2012
AF	p.E413K	Pathogenic	Pruszczyk et al., 2007
AF	p.E439K	Pathogenic	Wahbi et al., 2012
AF	p.R454W	Pathogenic	Wahbi et al., 2012
AF	p.E457V	Pathogenic	Hong et al., 2011
AF	p.D214-E245del	Pathogenic	Brodehl et al., 2021
AF	p.N342D	Pathogenic	van Spaendonck-Zwarts et al., 2012
AF	p.S13F	Pathogenic	Abou Ziki et al., 2021
AVB	p.S12F	Pathogenic	Hong et al., 2011
AVB	p.R16C	Pathogenic	Sharma et al., 2009
AVB	p.E234K	Likely pathogenic	Chen et al., 2017
AVB	p.E245D	Pathogenic	Conover et al. (2009)
AVB	p.L274P	Pathogenic	Hong et al., 2011
AVB	p.N342D	Pathogenic	Wahbi et al., 2012
AVB	p.I367F	Pathogenic	Olivé et al., 2007
AVB	p.R406W	Pathogenic	Arbustini et al., 2006
AVB	p.R454W	Pathogenic	Wahbi et al., 2012
AVB	p.E457V	Pathogenic	Hong et al., 2011
AVB	p.S460I	Pathogenic	Chourbagi et al., 2011
AVB	p.X471Y	Pathogenic	Chourbagi et al., 2011
AVB	p.E410K	Pathogenic	Fischer et al., 2021
AVB	p.R355P	Pathogenic	Wahbi et al., 2012
AVB	p.Y112H	Pathogenic	Brodehl et al., 2019
LBBB	p.K144X	Pathogenic	Wahbi et al., 2012
LBBB	p.S298L	Likely pathogenic	Olivé et al., 2007
LBBB	p.E413R	Pathogenic	Wahbi et al., 2012
LBBB	p.I402T	Pathogenic	Fischer et al., 2021
RBBB	p.E245D	Pathogenic	Strach et al., 2008
RBBB	p.A337P	Pathogenic	Goldfarb et al., 1998
RBBB	p.L345P	Pathogenic	Sjöberg et al., 1999
RBBB	p.S13F	Pathogenic	van Spaendonck-Zwarts et al., 2012
LAFB	p.E108K	Pathogenic	Taylor et al., 2007
ARVC	p.S13F	Pathogenic	van Tintelen et al., 2009
ARVC	p.N116S	Pathogenic	Klauke et al., 2010
ARVC	p.P419S	Pathogenic	Otten et al., 2010
ARVC	p.N342D	Pathogenic	Hedberg et al., 2012

AF, atrial fibrillation; AVB, atrioventricular block; LAFB, left anterior fascicular block; LBBB, left bundle-branched block; RBBB, right bundle-branched block; ARVC, arrhythmogenic right ventricular cardiomyopathy.

## Pathophysiological mechanisms of desmin variant-induced cardiac conduction disease and arrhythmia

By utilizing *DES* variant model systems, the most obvious pathological hallmark for cardiac dysfuntion is an abnormal cytoplasmic configuration of the desmin network and desmin aggregation formation. Desmin aggregation has been observed in subsarcolemmal, intermyofibrillar, and perinuclear regions (Carlsson et al., 2002). In mice overexpressing desmin with a seven amino acid deletion, toxic aggregates of desmin were observed. As desmin normally interacts with other cytoskeletal proteins and organelles, loss in this network will disrupt the continuity and overall organization of cell structure from the sarcolemma to the nuclear envelope (Wang et al., 2001). One patient with a homozygous missense mutation in the *DES* gene (p.Y122H) was diagnosed with restrictive cardiomyopathy and AVB. By generating an induced pluripotent stem cell (iPSC) model expressing p.Y122H in combination with functional analysis, Brodehl et al. (2019) showed that variant p.Y122H caused severe filament assembly defect and desmin aggregation, which may drive AVB.

**FIGURE 4:**
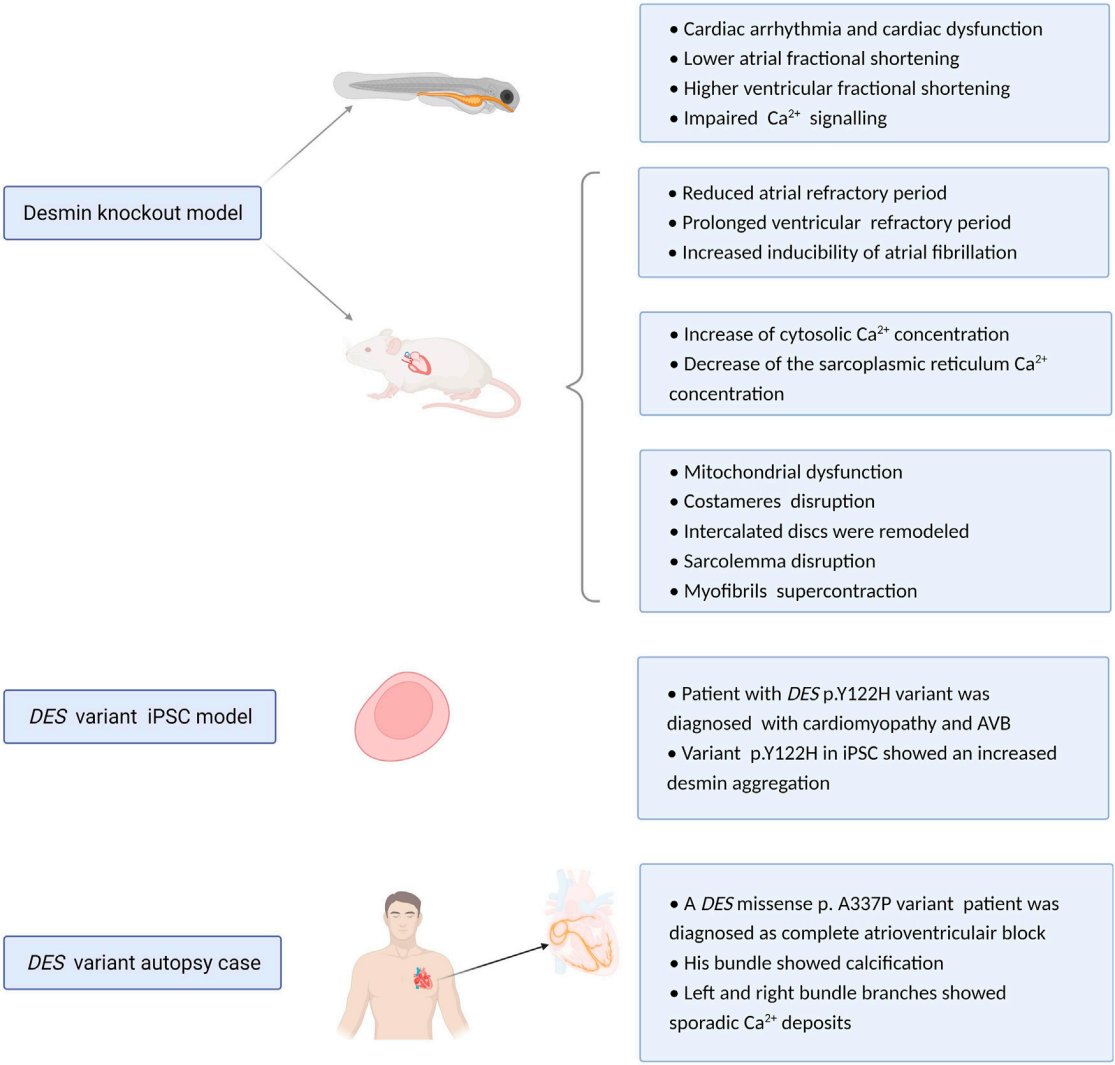
Overview potential pathophysiological mechanisms related to *DES* variants or loss. Experimental desmin knockout and variant model systems reveal a role for desmin impairment in development of cardiac conduction defects and arrhythmias. Proposed mechanism of action is via altered refractory periods, cytosolic Ca^2+^ concentrations, loss in desmin network, protein aggregation, and calcification.

Regarding cardiac conduction diseases, AVB may be associated with anatomical interruption of the atrioventricular conduction system. This pathological phenotype has been reported in the cardiac conduction system of one patient with p.A337P variation in the *DES* gene (Figure 4). This is an autopsy case from a Japanese man who showed His bundle calcification and left and right bundle branches with sporadic calcium deposits (Yuri et al., 2007). Conversely, another autopsy case from Brazil showed no calcification of the atrioventricular junction and His bundle, there was extensive fibrosis of the terminal portions of the branching bundle and the beginning of the left and right bundles at the top of the ventricular septum (Benvenuti et al., 2012). Further studies are needed to elucidate the role of desmin disruption, calcification and onset of AVB.

## Experimental desmin knock out models and diversity in cardiac arrhythmias

Previous studies utilizing *DES* knockout mouse models showed that these mice develop and reproduce normally and display no obvious anatomical defects (Li et al., 1997). However, ultrastructural studies of heart tissue samples from *DES* knockout mice reveal damage including swollen, disintegrated and abnormal distribution of mitochondria, all features that are indicative of mitochondrial dysfunction (Agbulut et al., 2001). Furthermore, costameres were found disrupted, especially at Z-domains (O'Neill et al., 2002). Five days post-partum, cardiomyofibers degenerate and 10 days post-partum, this was complemented with an accumulation of macrophages, fibrosis, and calcification of the inter-ventricular septum and the free wall of the right ventricle (Sprinkart et al., 2012). Also, at the ultrastructural level, intercalated discs were remodeled, sarcolemma disruption and myofibrils showed super contraction phenotype (Thornell et al., 1997).

In electrocardiography studies, desmin deficient mice present a significantly reduced atrial but prolonged ventricular refractory period, indicating increased inducibility of atrial arrhythmias but diminished susceptibility to ventricular arrhythmias (Schrickel et al., 2010). Moreover, knockdown of desmin in cardiomyocytes results in an abnormal distribution of Ca^2+^. Here, a marked increase in cytosolic Ca^2+^ concentration and a decrease in the sarcoplasmic reticulum Ca^2+^ concentration was found. As cytosolic Ca^2+^ overload is a trigger for AF (Brundel et al., 2002a; Brundel et al., 2002b) and Ca^2+^ is a critical element in the electrical excitation of cardiomyocytes, abnormal Ca^2+^ homeostasis may represent a mechanism by which desmin loss participates in cardiac arrhythmia (Chen et al., 2020).

Compared with mouse models, utilization of zebrafish as a model systems is easier to analyze the effect of desmin knock out on desmin aggregation in the heart. Ramspacher et al. (2015) generated two zebrafish models to compare a desmin loss of function model with desmin aggregate formation model. They found that both models led to cardiac arrhythmia and cardiac dysfunction. These defects are related to abnormal Ca^2+^ flux due to the disruption of excitation-contraction coupling machinery and abnormal subcellular localization of ryanodine receptor (Figure 4).

## Future therapies for desmin-induced cardiac arrhythmias

Desmin aggregation in cardiomyocytes is the most significant histopathological hallmark of desmin cardiomyopathies. Moreover, desmin aggregation leads to cardiomyopathy phenotypes (Ramspacher et al., 2015). One of the critical steps for a future therapeutic approach to desmin cardiomyopathies is to characterize representative animal models (e.g., mice, zebrafish) that phenocopy desmin aggregation in patients. Small heat shock proteins, including HSP27, αA-crystallin, αB-crystallin, and HSP22 prevent protein accumulation and aggregation formation (Garrido et al., 2012). One previous study showed that the non-toxic HSP inducer geranylgeranylacetone (GGA), a nontoxic antiulcer drug and inducer of small HSPs can inhibit desmin-related cardiomyopathy progression (Sanbe et al., 2009). This study indicated that GGA can induce expression of HSPB8 and HSPB1 inhibit protein aggregation. GGA led to a reduction in heart size and inhibition of interstitial fibrosis, and recovery of cardiac function as well as improved survival (Sanbe et al., 2009). Additionally, manipulating cell signaling pathways (i.e., PAK1, Rac1, PKC, or NSC23766), activating autophagy (mTOR inhibitor PP242), and using antioxidants (α-tocopherols or trolox) efficiently reduces up to 75% of aggregation of desmin variants in muscle cells (Cabet et al., 2015).

Targeting *DES* gene regulation could be a kind of effective treatment that leads to a decreased expression of the mutant *DES* allele. Such as RNA-targeted therapeutics, and methods based on DNA genome editing using CRISPR-Cas9 (Abdelnour et al., 2021). Nicorandil, a vasodilatory drug, was shown to prevent ventricular tachyarrhythmias induction by normalizing Cx43 expression in this desmin-related cardiomyopathy mouse model (Matsushita et al., 2014).

Although there are no clear and effective treatment methods and drugs for impaired desmin-induced cardiac arrhythmia, some complications can be prevented. For patients with early onset cardiac arrhythmia, early whole genome sequencing, 24-hour holter monitoring, and treatment of cardiac arrhythmias and conduction defects are important, since early diagnosis allows to make treatment plans at an early stage, reducing the incidence of serious heart failure. At the same time implantation of a pacemaker can be lifesaving. Modulating mitochondrial function such as nicotinamide mononucleotide, mitochondria-targeted peptides, gene, and stem-cell therapy or muscle-specific gene transfer approaches are active areas of research that promise effective treatments in the future (Goldfarb et al., 2004; Clemen et al., 2013).

## Summary

The desmin network plays a significant role in maintaining the structural and mechanical integrity of cardiomyoctyes. Moreover, desmin is involved in cardiomyocyte function by modulating cellular signaling and calcium homeostasis. As desmin is highly expressed in structures of the cardiac conduction system, the majority of pathogenic *DES* variants cause cardiac arrhythmia and cardiac conduction defects. On the molecular level, impaired desmin causes severe filament assembly defects, desmin aggregation, and abnormal distribution of Ca^2+^, that collectively may drive cardiac arrhythmias and cardiac conduction defects. Finally, further studies should elucidate the exact molecular mechanism how desmin affects specific cardiac conduction system structures. This knowledge will aid in the identification of druggable targets which may fuel development of effective mechanism-based therapies to treat cardiac conduction defects and arrhythmias.
